# Reconstructing brain functional networks through identifiability and deep learning

**DOI:** 10.1162/netn_a_00353

**Published:** 2024-04-01

**Authors:** Massimiliano Zanin, Tuba Aktürk, Ebru Yıldırım, Deniz Yerlikaya, Görsev Yener, Bahar Güntekin

**Affiliations:** Instituto de Física Interdisciplinar y Sistemas Complejos IFISC (CSIC-UIB), Campus UIB, Palma de Mallorca, Spain; Program of Electroneurophysiology, Vocational School, Istanbul Medipol University, Istanbul, Turkey; Health Sciences and Technology Research Institute (SABITA), Istanbul Medipol University, Istanbul, Turkey; Department of Neurosciences, Health Sciences Institute, Dokuz Eylül University, Izmir, Turkey; School of Medicine, Izmir University of Economics, Izmir, Turkey; Brain Dynamics Multidisciplinary Research Center, Dokuz Eylül University, Izmir, Turkey; Department of Biophysics, School of Medicine, Istanbul Medipol University, Turkey

**Keywords:** EEG, Deep learning, Functional networks, Alzheimer’s disease, Parkinson’s disease

## Abstract

We propose a novel approach for the reconstruction of functional networks representing brain dynamics based on the idea that the coparticipation of two brain regions in a common cognitive task should result in a drop in their identifiability, or in the uniqueness of their dynamics. This identifiability is estimated through the score obtained by deep learning models in supervised classification tasks and therefore requires no a priori assumptions about the nature of such coparticipation. The method is tested on EEG recordings obtained from Alzheimer’s and Parkinson’s disease patients, and matched healthy volunteers, for eyes-open and eyes-closed resting–state conditions, and the resulting functional networks are analysed through standard topological metrics. Both groups of patients are characterised by a reduction in the identifiability of the corresponding EEG signals, and by differences in the patterns that support such identifiability. Resulting functional networks are similar, but not identical to those reconstructed by using a correlation metric. Differences between control subjects and patients can be observed in network metrics like the clustering coefficient and the assortativity in different frequency bands. Differences are also observed between eyes open and closed conditions, especially for Parkinson’s disease patients.

## Author Summary

Functional networks are becoming a standard tool in neuroscience, thanks to their ability to extract the interactions between brain regions and representing them as simple mathematical objects. Many metrics for reconstructing these networks have so far been proposed, based on different assumptions about how those interactions manifest in the recorded data, and consequently depicting a limited part of the picture. We here introduce a more general approach, based on the idea that brain regions should have a unique dynamic, which becomes more similar when they coparticipate in a cognitive task, and on the quantification of such uniqueness through deep learning models.

## INTRODUCTION

In spite of many decades of experimental and theoretical works, the principles and mechanisms behind brain activity during complex cognition tasks or even simple resting conditions remain elusive; this is even true when considering conditions that strongly alter the normal dynamics, as, for example, in Alzheimer’s or Parkinson’s diseases. One possible avenue for improving our understanding of the brain is network neuroscience (Bassett & Sporns, 2017; de Vico Fallani, Richiardi, Chavez, & Achard, 2014; Fornito, Zalesky, & Bullmore, 2016; Sporns, 2014, 2018), that is, the representation of brain activity through networks, in which nodes represent different brain regions, and pairs of them are connected whenever an information propagation is detected between the corresponding regions. This approach is explicitly integrative, as elements are characterised by their connectivity, and merges empirical data acquisition with computational approaches, therefore being at the intersection of biomedicine and computer science. Most importantly, it has yielded groundbreaking insights on how different pathologies affect the normal brain dynamics (Calhoun, Eichele, & Pearlson, 2009; Fleischer et al., 2019; Sala-Llonch, Bartrés-Faz, & Junqué, 2015; van Diessen, Diederen, Braun, Jansen, & Stam, 2013; L. Wang et al., 2009).

One of the underlying assumptions of this network approach is that the coparticipation of two or more brain regions in a computational task results in some common characteristics in the corresponding regions’ dynamics. In other words, brain regions have a unique dynamics when isolated, but by participating in a task (e.g., by sharing some information), such different dynamics become more similar. The simplest example is provided by the reconstruction of functional networks through linear correlations, where the commonality is expected to be a correlated amplitude across regions. Similar hypotheses are made when more sophisticated metrics are used, like, for instance, causality (a relationship between the past of the causing time series and the future of the caused one) (Ding, Chen, & Bressler, 2006; Seth, Barrett, & Barnett, 2015) or synchronisation metrics (a relationship between the phases of the signals) (Heitmann & Breakspear, 2018; Varela, Lachaux, Rodriguez, & Martinerie, 2001). In other words, reconstructing functional networks is akin to detecting emerging (*lato sensu*) similarities.

In this contribution we explore the opposite approach, that is, how coparticipation in cognitive tasks can be detected as a drop in the uniqueness of the dynamics of the involved brain regions. In other words, not dissimilarly from what assumed in standard functional network reconstruction, we suppose that brain regions are functionally homogeneous and characterised by unique dynamics (Korhonen, Saarimäki, Glerean, Sams, & Saramäki, 2017); such uniqueness is nevertheless partly lost when performing a shared cognitive task. Thus, instead of detecting an increased similarity, we shift the focus towards a reduced uniqueness. Far from being a mere semantic transformation, this approach entails two important advantages. First of all, it makes no assumptions on what is shared by the brain regions’ dynamics, for example, correlation, phase, and so forth. On the contrary, any common aspect able to reduce the uniqueness of the dynamics is taken into consideration, provided, of course, that we have means to detect such reduction. Secondly, this approach can be framed as a classification task, in which a machine learning model is trained to recognise time series recorded from two brain regions; the higher the classification score, the more unique (or identifiable) the two regions are, and hence the less they are participating in the same cognitive tasks. In turn, this allows us to resort to deep learning (DL) models (Goodfellow, Bengio, & Courville, 2016; LeCun, Bengio, & Hinton, 2015; Rusk, 2016), that is, state-of-the-art machine learning algorithms not assuming nor requiring a priori structures in the data, and extremely sensitive, that is, able to detect even subtle and complex differences between datasets.

While DL models are not new to neuroscience (De Schutter, 2018; Marblestone, Wayne, & Kording, 2016; Saxe, Nelli, & Summerfield, 2021; Vogt, 2018), our approach is different in two major aspects. First of all, classification models are not used to actually classify, that is, to predict the label of a given set of data, as when one tries to discriminate between control subjects and patients (Oh et al., 2020; Ortiz, Munilla, Gorriz, & Ramirez, 2016). On the contrary, the score of the classification task is used to assess the uniqueness of each set of data, that is, its identifiability. Secondly, the result of the classification is not used in an isolated fashion, but is instead only a step in a more complex analysis based on network representations. The classification is thus not the aim, but the instrument of the analysis.

We here explore the possibilities and limitations of this approach, by applying it to a dataset of EEG resting-state recordings of Parkinson’s (PD) and Alzheimer’s (AD) disease patients and matched control subjects. We first discuss some technical aspects, including the available DL algorithms for performing supervised classification of time series and the tuning of the models’ parameters. We then move to data analysis, by quantifying the identifiability of pairs of channels in the three conditions and the topological properties of the resulting functional networks. We conclude by drawing some considerations about the advantages and drawbacks of this approach, and sketching some possible lines for future research.

## MATERIALS AND METHODS

### Control Subjects and Patients Recruiting and Selection

Fifty participants were recruited in this study, divided into three groups according to their health condition: AD and PD patients, and healthy elderly control subjects. Subjects’ demographic information is presented in Table 1. Complete neurological examination, structural magnetic resonance imaging (MRI), routine screening laboratory examinations, and an extensive battery of neuropsychological tests were applied to all participants.

**Table 1.** Demographic data of the subjects analysed in this study

**Subject group**	**Size**	**Of which men/women**	**Avg. age (*STD*)**	**Avg. years of education (*STD*)**
Control	19	11/8	69.1 (7.25)	10.9 (4.67)
AD	19	5/14	73.2 (5.68)	8.8 (4.40)
PD-D	12	9/3	73.0 (6.71)	5.9 (5.21)

The healthy elderly control subjects with no neurological abnormality were included in the study when no global cognitive impairment (Mini-mental State Examination (MMSE) score ≥27) was determined.

The probable PD-D diagnosis was made according to the Movement Disorder Society (MDS) Level 1 criteria (Dubois et al., 2007; Emre et al., 2007). Probable PD-D diagnosis was made when all the following five criteria were met: (i) PD diagnosis according to United Kingdom Parkinson’s Disease Society Brain Bank Criteria (Hughes, Daniel, Kilford, & Lees, 1992); (ii) development of PD prior to the onset of dementia; (iii) PD associated with a decreased global cognitive score that was defined as a score of ≤24 on the MMSE (Folstein, Folstein, & McHugh, 1975); (iv) cognitive impairment that causes dysfunction in daily life; and (v) cognitive impairments found in more than one domain.

All individuals with Alzheimer’s disease dementia (AD) were diagnosed according to the National Institute of Aging Alzheimer’s Association diagnostic guideline (McKhann et al., 2011). The inclusion criteria for AD dementia patients included impairment in two or more cognitive domains and impaired daily living activities with CDR score of 1 or 2. The exclusion criteria for AD dementia patients included history or presence of any other psychiatric and/or neurological disorders including traumatic brain injury, depression, alcohol or drug abuse, and vascular brain lesions. All individuals with AD dementia were using cholinesterase inhibitor medications (donepezil: 5–10 mg per day; and rivastigmine: 6 mg–9.5 mg/24h per day), and some patients were in addition on memantine (10–20 mg per day).

The exclusion criteria for all participants included (i) history of neurological and/or psychiatric disorders, including evidence of depression as demonstrated by Yesavage Geriatric Depression Scale scores higher than 13 (Ertan, Eker, Şar, et al., 1997; Yesavage et al., 1982); (ii) presence of any uncontrolled medical illnesses; (iii) history of severe head injury and abusive drug or alcohol usage; (iv) using any psychoactive drugs or cognitive enhancers (besides acetylcholinesterase inhibitors and memantine for AD patients); and (v) presence of structural brain lesions including vascular brain lesions with Fazekas score equal or greater than 3, hydrocephalus, or any brain tumor in MRI.

The study is designed according to the principles of the Declaration of Helsinki. All participating individuals and/or their relatives gave written informed consent for the study, that was approved by the local ethical committee (Istanbul Medipol University Ethical Committee, Report No: 10840098-604.01.01-E.8374).

### Electroencephalographic Data Recording

EEG of the subjects was recorded with a Brain Products BrainAmp 32 Channel DC system with the band limits of 0.001–250 Hz and 500 Hz sampling rate. EasyCap was used with 32 Ag/AgCl electrodes that were placed according to the 10/20 system, and all electrode impedances were kept below 10kΩ. A1 and A2 electrodes were used as reference and placed on the earlobes. In order to record the Electrooculogram, two Ag/AgCl electrodes were used, and they were placed on the left eyes’ medial upper and lateral orbital rim. The EEG was recorded in a dimly isolated room in two different centres with identical recording equipment, and the EEG recording procedure was applied precisely in the two centres. One center was Istanbul Medipol University REMER, Clinical Electrophysiology, Neuroimaging, and Neuromodulation Laboratory, and the other was Dokuz Eylül University Multidisciplinary Brain Dynamics Research Center. The EEG of all subjects was recorded as 4 minutes eyes open and 4 minutes eyes closed session (i.e., approximately 240,000 data points per channel and subject). During the eyes open recording session, the subjects were asked to look at the black screen. The subjects were monitored with a video camera during EEG recordings, and the researcher watched the subjects during all recording sessions. Beyond a basic manual analysis to discard recordings with evident artefacts and errors, no additional data preprocessing has been carried out, and, unless otherwise specified, broadband signals have been used.

### Deep Learning Models for Time Series Classification

This contribution leverages on the idea that the coparticipation in a cognitive task by two brain regions can be estimated through the drop in their corresponding identifiability, that is, how easy it is to recognise the corresponding EEG signals. This is performed by calculating the score obtained by a classification model, as large scores imply that the pairs of signals are different and easy to identify. Within the numerous models available in machine learning for time series classification (Abanda, Mori, & Lozano, 2019; Bagnall, Lines, Bostrom, Large, & Keogh, 2017), we here resort to deep learning, that is, a set of machine learning algorithms that progressively extract higher level features from the raw input (Deng & Yu, 2014; LeCun et al., 2015). Compared to standard machine learning models, DL ones present the advantage of not assuming nor requiring a priori structures in the data, and of not requiring a preprocessing of features; in other words, features are automatically extracted from data, without human intervention. This results in a drastically higher efficiency, especially in complex problems for which features are difficult to be defined. On the other hand, this also implies high computational costs, and usually the need of dedicated hardware as, for example, general purpose graphics processing unit (GPGPU).

In agreement with the objectives of the contribution, we here focus on time series classification models; in other words, given a set of time series, each one associated with a label (i.e., the EEG channel it corresponds to), the objective is to assign the correct label to a new time series presented to the algorithm. Several models for this task have been proposed in the literature, usually evolutions of models designed for image classification (see Fawaz, Forestier, Weber, Idoumghar, & Muller, 2019, for a full review, and https://github.com/hfawaz/dl-4-tsc for the corresponding source codes). More specifically, the following five models have been used:

- *Multi layer perceptron (MLP)*. One of the most traditional and simplest forms of neural network, it is composed of a set of nodes organised in layers, each one receiving information from the previous layer and responding through a nonlinear activation function. Even though it does not encode temporal information, the MLP model has been proposed as a baseline architecture for classifying time series (Z. Wang, Yan, & Oates, 2017). The network here considered is composed of four layers, each one fully connected to the outputs of the previous one, and with the final layer being a *softmax* classifier. The activation function is the well-known rectifier linear unit (ReLU) (Nair & Hinton, 2010)
- *Convolutional neural network (CNN)*. Convolutional networks are specialised versions of MLP, in which the matrix multiplication is substituted by a convolution operation (Albawi, Mohammed, & Al-Zawi, 2017). Their advantages include a space (or, in the case of time series, time) invariance (W. Zhang et al., 1988), and a reduced tendency to overfitting. We here consider a simple convolutional model, composed of two convolutional layers followed by a final *sigmoid* classifier.
- *Residual network (ResNet)*. Residual networks are inspired on the way pyramidal cells are organised in the cerebral cortex; specifically, the connections between layers are not sequential, but instead some layers can be skipped (creating shortcuts or jumps). This presents the advantage of a simpler structure, and consequently of a reduced training cost (He, Zhang, Ren, & Sun, 2016). The networks here considered are composed of 11 layers, the first 9 of them being convolutional, followed by a global average pooling layer that averages the time series across the time dimension, and by a final *softmax* classifier, as proposed in Z. Wang et al. (2017).
- *Fully convolutional neural network (FCN)*. FCNs are networks in which only convolution operations can be performed; in other words, they are equivalent to CNNs without fully connected layers (Long, Shelhamer, & Darrell, 2015). The model is composed of three convolutional blocks, each one performing a convolution, a batch normalisation, and a final activation. As a last step, the result of the third convolutional block is fed to a *softmax* classifier (Z. Wang et al., 2017).
- *Multi channel deep convolutional neural network (MCDCNN)*. This model is based on a modified CNN, in which the convolutions are applied independently (in parallel) on each dimension (or channel) of the input multivariate time series (Zheng, Liu, Chen, Ge, & Zhao, 2014, 2016). As here only univariate data are considered, that is, each EEG channel is described by a single time series, results of MCDCNN and CNN are expected to be similar albeit not always the same; see Supporting Information for a comparison of the two.

While these five models are not as complex as others that can be found in the literature, as for example, with respect to the number of layers, they still contain a number of parameters well beyond what found in classical machine learning, from ≈100 for MLP, to ≈10^6^ in the case of ResNet. Additionally, they generally achieve good classification scores, as will be discussed below. The five models have been implemented in Python 3.8.5 using the libraries TensorFlow (Abadi et al., 2016) and Keras (Gulli & Pal, 2017).

### Classification Tasks and Identifiability

Given the time series of a pair of EEG channels for all subjects belonging to a group (control subjects, and Alzheimer’s and Parkinson’s disease patients, see below), the identifiability of those two channels has been assessed as the best classification score obtained by a DL model. From a mathematical perspective, each analysis starts with a dataset {*X*, *Y*}_*c*_, where *X* denotes a set of time series, *Y* the corresponding labels (i.e., the EEG sensor from which the time series have been recorded), and *c* the group (here control, AD or PD). The classification model is then a function *f*(*x*, *θ*) that, given a new time series *x* not included in *X* and a set of parameters *θ*, yields the best estimation of the label *y* associated to *x*. *θ*, representing the internal weights of the DL model, are obtained in the training phase to minimise the error between the estimated class $\hat{Y}$ = *f*(*X*, *θ*) and the real one *Y*. In short, the objective of the task is to recognise the EEG sensor from which a given time series has been recorded, using other examples as a reference.

In each iteration of the classification problem, the time series have been split into nonoverlapping segments of 1,000 points (equivalent to 2 seconds). Note that such length has arbitrarily been chosen, as a compromise between the need of long segments to achieve meaningful classifications, and of having enough segments for training the models; a full discussion on this problem is included in Supporting Information. A random half of the available time series has been used for training, and the remaining half for evaluating the model, being thus equivalent to a two-fold cross-validation. Each model performance is finally measured through the corresponding accuracy score, that is, the fraction of correctly classified segments; note that other complementary metrics, for example, recall or F-score, are here redundant due to the use of a perfectly balanced dataset. For the sake of clarity and in order to avoid confusions with the complex network metric of the same name, the efficiency will here be referred to as classification score, or simply as score in figures where enough space is not available.

It is worth noting that the problem here described is similar to, but not the same as, identifiability as usually conceived in neuroscience, that is, the evaluation of individual fingerprints in the connectivity structure. While previous works have focused on identifying the subject behind a given functional network (Abbas et al., 2020; Amico & Goñi, 2018; Bari, Amico, Vike, Talavage, & Goñi, 2019; Rajapandian, Amico, Abbas, Ventresca, & Goñi, 2020; Sorrentino et al., 2021; Van De Ville, Farouj, Preti, Liégeois, & Amico, 2021), in a problem conceptually similar to identity assurance, we here tackle the identification of the source of an EEG signal, and use such identifiability to reconstruct the network representation. Similarly, this work departs from the large body of literature using DL models to classify subjects (Al-Saegh, Dawwd, & Abdul-Jabbar, 2021; Craik, He, & Contreras-Vidal, 2019). The aim here is not to create a model able to assign a label to each subject, as such label (control subject, AD or PD patient) is initially given, but rather to describe the relationships between the signals recorded by pairs of EEG sensors, as a function of the group each person belongs to.

The previously described DL models are based on different architectures, and thus their effectiveness in detecting different patterns in the data can vary. This may result in different classification scores for the same task but, additionally, in the best model changing depending on the considered couple of EEG channels. As here we are interested in the identifiability of pairs of channels, independently on the patterns supporting such identifiability, the score obtained by the best model in each classification problem (i.e., for each pair of EEG channels) has been selected.

### Model Hyper-parameters’ Tuning

As true for any other machine learning algorithm, DL models require setting some hyper-parameters to optimise the outcome of the classification. The first one is the number of epochs, that is, the number of times the training is performed over all available data. This number controls a trade-off: the larger the number of epochs, the more accurate the classification is expected to be, albeit at an increased computational cost. Furthermore, beyond a certain level, performing additional trainings yields no further improvements.

Secondly, one has to note that the training is a stochastic process: the original data are split between training and validation sets at random, and the initial state of the neural network is also random. As a consequence, it is customary to repeat the whole training and validation process several times, and to finally average the result. The second hyper-parameter is thus the number of iterations, that is, executions of the full training and evaluation cycle with random initial conditions. The larger this value, the more stable the final result is, yet again at the price of a larger computational cost.

**Figure 1.**
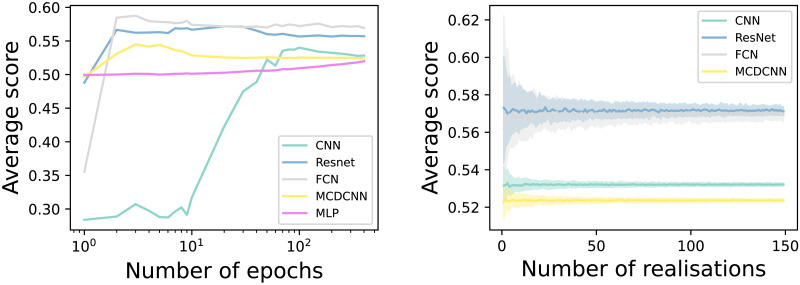
Tuning DL models’ hyper-parameters. (Left) Evolution of the average classification score, for channels Fz and Cz and control subjects, as a function of the number of epochs in the training. (Right) Evolution of the 10–90 percentile range for the same classification problem, as a function of the number of realisations.

Figure 1 reports the results of tuning these two hyper-parameters. Specifically, we have considered one pair of EEG channels (Fz and Cz), and performed the corresponding classification varying them. The two channels have been selected for presenting few artefacts, and due to their proximity, they have similar dynamics and are therefore difficult to classify. The left panel of Figure 1 reports the evolution of the average score obtained by each model, as a function of the number of epochs. Some models, for example, MCDCNN, only require a few epochs, while CNN only saturates for hundreds of epochs. As a compromise between precision and computational cost, the following numbers of epochs have been selected: 200 for CNN, and 20 for all others. Additionally, MLP has been discarded due to being substantially worse than the other models.

For the remaining four models, the right panel of Figure 1 reports the evolution of the 10–90 percentile of the obtained score, as a function of the number of realisations of the classification task. One hundred iterations is enough to obtain very stable results with all models.

### Functional Network Reconstruction and Analysis

As standard in complex network theory, a network is fully defined by its adjacency matrix *A*, of size *N* × *N* (*N* being the number of nodes), whose element *a*_*i*,*j*_ is set to 1 when a link connects nodes *i* and *j*, and to 0 otherwise (Boccaletti, Latora, Moreno, Chavez, & Hwang, 2006; Strogatz, 2001). In the network neuroscience context, *a*_*i*,*j*_ is then set to 1 when the synchronisation metric (e.g., correlation) calculated between the time series corresponding to sensors *i* and *j* is larger than a given threshold, as this indicates the presence of a shared dynamics. As previously introduced, the approach here proposed is the opposite: as a shared dynamics will result in a reduction in the identifiability of those two sensors, a link is added whenever such identifiability (as measured through the corresponding classification score) is below the threshold. In other words, the lower the classification score, the stronger the connection between the corresponding brain regions is expected to be.

Once these networks have been reconstructed, they are binarised, that is, links whose strength is above a given threshold are retained and all other are deleted. This is a standard approach in network neuroscience, as allows to reduce the influence of weak (and hence, noisy) links (Van Wijk, Stam, & Daffertshofer, 2010). We here adopt a proportional thresholding approach, by including in each network a fixed number of the strongest links (van den Heuvel et al., 2017). This approach is often referred to in literature as an analysis in which the density (Jalili, 2016) or network cost (Achard & Bullmore, 2007) is kept constant, and has been suggested to be advantageous for case-control studies (Nichols et al., 2017; Váša, Bullmore, & Patel, 2018). Note that alternatives are available, most notably the absolute thresholding, which are here not evaluated.

The binarised networks are finally described by a set of standard topological metrics; these are shortly described below for the sake of completeness; the interested reader can find more detailed explanations in the literature (Bullmore & Sporns, 2009; Costa, Rodrigues, Travieso, & Villas Boas, 2007; Rubinov & Sporns, 2010).

- Clustering. Also known as transitivity, measures the presence of triangles in the network (Newman, 2003).
- Efficiency. The efficiency of a network represents how easily information can move between its nodes, and is defined as the inverse of the harmonic mean of the distances between pairs of nodes (Latora & Marchiori, 2001).
- Assortativity. Pearson correlation coefficient of the degrees of nodes at either ends of each links of the network (Noldus & Van Mieghem, 2015).
- Information content. Metric assessing the presence of regularities in the adjacency matrix of the network, that is, of mesoscale structures. It is calculated as the amount of information encoded in the adjacency matrix, such that small values correspond to regular topologies, and large values to random-like structures (Zanin, Sousa, & Menasalvas, 2014).
- Maximum degree. Degree (i.e., number of links) of the most connected node in the network.
- Rich club coefficient. Quantitative assessment of the rich club tendency of the network, according to which nodes with high centrality, that is, the dominant elements of the system, tend to form tightly interconnected communities (Colizza, Flammini, Serrano, & Vespignani, 2006).
- Modularity. Metric assessing the presence of a community structure in the network, that is, of groups of nodes densely connected between them, but loosely connected with other communities (Fortunato, 2010). The modularity is here calculated using the well-known Louvain algorithm (Blondel, Guillaume, Lambiotte, & Lefebvre, 2008).
- Nestedness. Metric measuring the tendency for nodes to interact with subsets of the interaction partners of better connected nodes (Mariani, Ren, Bascompte, & Tessone, 2019).

An additional difference between standard functional networks and the ones here reconstructed has to be highlighted. While the standard approach yields one network per subject, here the result is a single network per group of subjects, that is, per condition. This presents the advantage of better capturing the structures induced by the pathology under study, without the need of managing intersubject variability. At the same time, it complicates the assessment of the statistical significance of results, a topic that will be discussed in the conclusions.

## RESULTS

### Comparing DL Models

**Figure 2.**
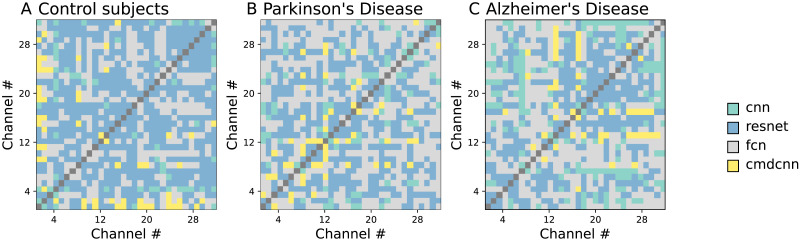
Comparison of the four DL models. Each panel reports the model yielding the best classification score for each pair of EEG channels; see right colour legend. Left, center, and right panels, respectively, correspond to control subjects and PD and AD patients.

We start the analysis of the results by comparing the score obtained by the different DL models; as previously discussed, these are based on different architectures and detect different patterns in the data, hence they are expected to yield heterogeneous results. For the three groups of subjects, Figure 2 reports the model yielding the best score for each pair of EEG channels. It can easily be appreciated that models distribute in a nonrandom fashion, with some models being associated to higher scores in some brain regions; see Supporting Information for a complete distribution by EEG channels, and a comparison of the results the models yield. In other words, brain regions are characterised by different activity patterns that are recognised more easily by some models. Additionally, there are differences across the three groups, suggesting that the local dynamics are further modified by the two pathologies.

### Identifiability of EEG Channel Pairs

**Figure 3.**
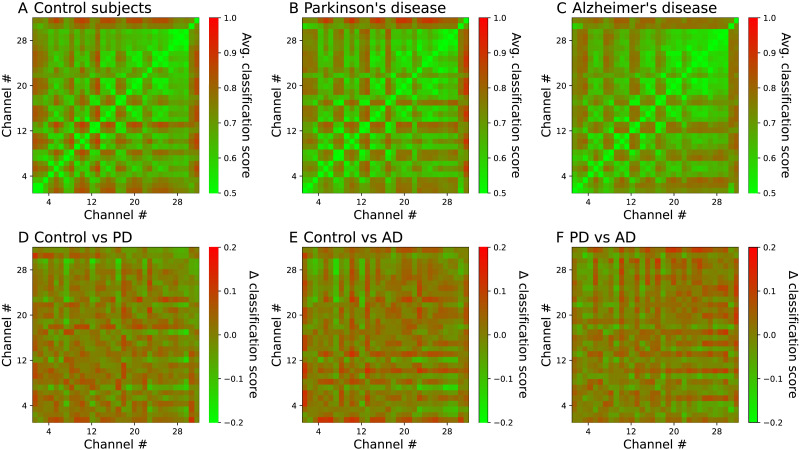
Identifiability of pairs of EEG channels. Top panels report the pairwise identifiability (i.e., the average classification score) between pairs of EEG channels, for the three conditions here considered. Bottom panels report the difference in identifiability between pairs of conditions.

We then move to the analysis of the identifiability of each pair of EEG channels. Specifically, the top three panels of Figure 3 report the average classification score obtained for each one of them, for the three conditions here considered. Note that, in the remainder of the work, the reported classification score for a given pairs of EEG channels corresponds to the one yielded by the best DL model, as identified in Figure 2. Results suggest that connectivity in the three groups shares the same underlying structure, and this is strongly influenced by crosstalk between sensors. In order to highlight condition-dependent patterns, the three bottom panels report the difference in identifiability between each pair of conditions, also by pairs of channels. Such difference is characterised by patterns centred in some specific EEG channels; see Supporting Information for an analysis by channels. More generally, identifiability is strongly reduced in AD patients when compared to control subjects; and, in less degree, in PD patients.

**Figure 4.**
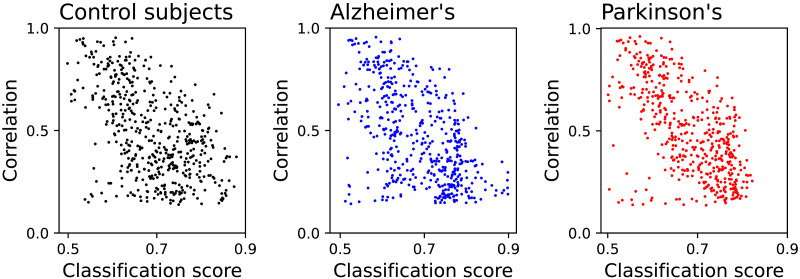
Linear correlation versus classification score. Each panel reports, for the three conditions here considered, a scatter plot of the average absolute linear correlation between pairs of channels, as a function of the corresponding classification score.

We further analyse two additional aspects of this pairwise identifiability. First of all, one may ask what is the relation between the identifiability, defined here as the score yielded by a classification task, and more traditional functional metrics. In order to answer this, Figure 4 reports three scatter plots, that is, one for each condition, of the average absolute value of the Pearson’s linear correlation between each pair of EEG channels, as a function of the corresponding classification score. As expected, a general negative correlation can be observed; in other words, highly correlated channels are more difficult to be recognised for being dynamically similar, and thus have a lower identifiability. Still, these two metrics are not the same; weakly correlated time series can yield both low and high classification scores, thus indicating that more complex patterns are detected by DL models. To illustrate, if the two time series have similar dynamics but with different characteristic frequencies, they will be easy to recognise, even though their correlation is low. On the other hand, two very complex time series will be weakly correlated, and also the identification of the patterns making them unique will be a challenging task. Note that a low correlation and identifiability can also be obtained in the case of highly noisy time series; this is nevertheless not the case at hand, as the EEG time series can easily be discriminated from their surrogate time series, and are thus not dominated by noise; see Supporting Information. Similar results for the comparison of the classification score with the Granger causality test (Ding et al., 2006; Seth et al., 2015) and the transfer entropy metric (Vicente, Wibral, Lindner, & Pipa, 2011), as well as the relationship between the score and the physical distance between sensors, are reported in Supporting Information.

**Figure 5.**
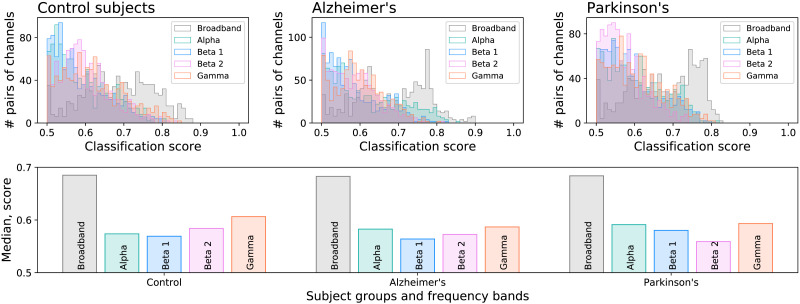
Identifiability and frequency information. Top panels report the histograms of the classification score, for the broadband signals, and signals filtered in four classical frequency bands. The bottom panel reports the median of the classification score, per condition and frequency band.

Secondly, we analyse how the identifiability depends on the frequency content of the time series. Specifically, the top panels of Figure 5 report the histograms of the classification scores, for each condition, and for broadband signals and four filtered bands: *α* (8–13 Hz), *β*_1_ (13–20 Hz), *β*_2_ (20–30 Hz), and *γ* (30–50 Hz). Additionally, the bottom panel of the same figure reports the median classification score for the three conditions and five frequency bands. Broadband time series are always more identifiable; small differences can be observed by conditions, for example, the drop in identifiability for PD patients and *β*_2_.

### Network Analysis

As previously introduced, and partly confirmed by Figure 4, the identifiability of pairs of EEG channels can be interpreted as a metric complementary to more classical alternatives used to reconstruct functional networks. In other words, two highly identifiable channels can be understood as the result of two brain regions functionally independent (or not cooperating in the cognitive task under analysis); on the other hand, a drop in identifiability can be due to a shared dynamics.

The usual way of reconstructing functional networks involves the pairwise comparison of pairs of EEG signals, for then obtaining one network for each subject under study. These networks, and more specifically the topological metrics from them extracted, can then be averaged across multiple patients to obtain a global picture of a given condition. While also possible in the case of the identifiability, we here explore a different alternative. Specifically, all time series corresponding to a pair of EEG channels are used for calculating the identifiability, that is, irrespectively of the specific subject for which have been recorded; the result is then a single network per condition.

**Figure 6.**
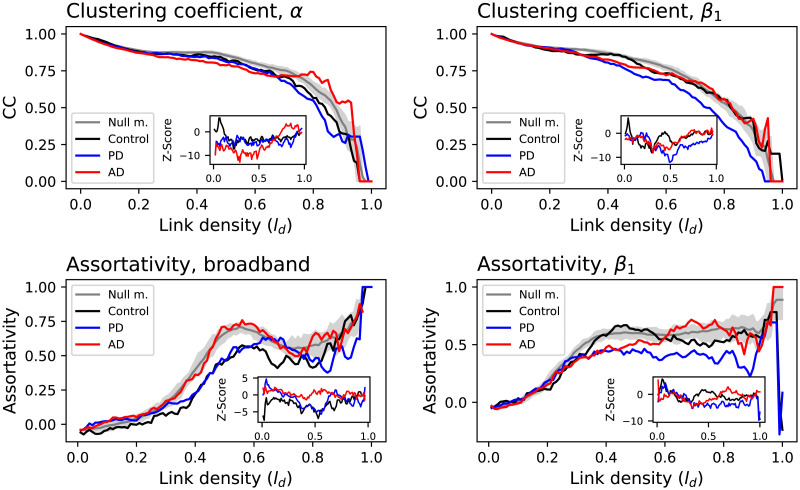
Examples of topological metrics. The four panels report examples of the evolution of the clustering coefficient (top) and assortativity (bottom), as a function of the link density. Insets report the corresponding metric evolution as Z-scores, calculated against a null model including all groups. See the Supporting Information for all topological metrics and frequency bands.

Following this idea, we have here reconstructed three functional networks, one for each condition, and have extracted the evolution of a set of standard topological metrics as a function of the link density. Four interesting examples are reported in Figure 6: the clustering coefficient, calculated for the *α* and *β*_1_ bands, and the assortativity for the broadband signal and the *β*_1_ band; see Supporting Information for results for all combinations of metrics and frequency bands. In order to evaluate the significance of those results, the grey bands report the 10–90 percentile of the same metrics, obtained through a null model based on reconstructing the functional networks including time series of all three groups; the insets inside each panel further report the evolution of the metric of each group as a Z-score, calculated against such null model. Some differences between groups can be observed. For instance, the clustering coefficient is higher for AD in the *α* band, but lower for PD in the *β*_1_ band; AD is also characterised by a larger assortativity with respect to PD, for the broadband signal and the *β*_1_ band. These differences are robust against reductions in the size of the dataset, as shown in Supporting Information.

**Figure 7.**
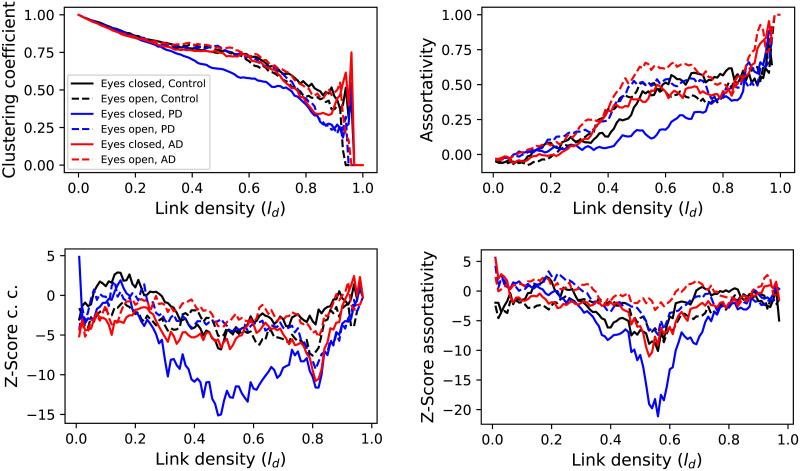
Comparison of eyes open and closed conditions. The top left and right panels, respectively, depict the evolution of the clustering coefficient and assortativity, as a function of the link density in the reconstructed networks, for the three conditions and eyes closed (solid lines) and open (dashed lines). The bottom panels depict the evolution of the same metrics as a Z-score, calculated against a null model comprising random segments from the three conditions. See the Supporting Information for all topological metrics.

We further analysed how these topological metrics are affected by the subjects having their eyes open or closed, by performing the classification task separately for the part of the time series corresponding to each condition. Figure 7 illustrates the evolution of two topological metrics, while full results are reported in the Supporting Information; the top panels in that figure correspond to the raw metric values, while bottom ones to a Z-score calculated using a null model, that is, the same as insets of Figure 6. Some general trends can be observed, as the fact that PD eyes closed and AD eyes open patients always display the greater difference: the former (latter) having the smallest (respectively, largest) clustering coefficient and assortativity, but largest (smallest) nestedness. The Supporting Information also reports the difference in identifiability by channel when comparing the two conditions, while in general the eyes open condition is associated with a higher identifiability; this is especially marked in the case of PD patients.

## DISCUSSION AND CONCLUSIONS

In this contribution we proposed a new methodology for analysing neuroimaging signals, based on the assessment of the identifiability, or “uniqueness,” of the dynamics of each brain region. Instead of making a priori assumptions about which aspects of the time series have to be analysed to detect similarities, this approach allows to reverse the question and to focus on any dynamical aspect that makes a time series, or a set of them, unique. This, in turns, allows to resort to deep learning, that is, the state of the art in machine learning models. The final result is a metric that can be used to detect the loss of individual dynamics caused by the cooperation in a cognitive task, and consequently a new way of reconstructing functional brain networks.

It may be tempting to assume that the identifiability here proposed is qualitatively similar to existing metrics to detect functional connectivity. While some correlation is indeed present (see Figure 4 and Supporting Information), they do not measure the same. To illustrate, it is well known that Alzheimer’s disease patients have a reduced resting-state functional connectivity (Dauwels, Vialatte, Musha, & Cichocki, 2010; C. Stam et al., 2009; C. J. Stam et al., 2006); we nevertheless here found the opposite, that is, that the identifiability of EEG channels of AD patients is lower than that of control subjects. Therefore, even if the dynamics of pairs of brain regions are less synchronised, they are not necessarily more different, or, in other words, they fail in developing a recognisable dynamics. The aforementioned difference in the identifiability between control subjects and AD patients is also observed in the case of PD patients. Still, it is important to note that such changes are not homogeneous, but rather distribute unevenly across sensors; to illustrate, F8 and TP7, respectively, have the larger increase and decrease of identifiability in PD patients, and F8 and FCz in AD ones; see Supporting Information. Additionally, conditions also modify which DL model achieves the best classification; see Figure 2.

At a microlevel, it can thus be concluded that DL models are detecting changes in the EEG time series, both in their uniqueness, and in the patters that support such uniqueness; it is nevertheless challenging, due to the black box nature of DL, to provide a description of those patterns. Moving to the macroscale, the reconstructed functional networks highlight some important differences between control subjects and patients. Specifically, AD patients are characterised by a larger clustering coefficient and assortativity, while lower values of both metrics can be observed for PD patients (see Figure 6). Also, important differences can be observed between the eyes-closed and open conditions for PD patients, with the former having lower values of clustering coefficient and assortativity (see Figure 7).

One of the advantages of the proposed method is that it allows reconstructing both individual functional networks per subject, and the global network representing a condition. While here not considered, a similar analysis could be performed using multiple time series (or segments thereof) recorded from a single subject, for then evaluating the identifiability of pairs of EEG sensors; the result would be an identifiability functional network for each subject, thus akin to the standard approach in network neuroscience. On the other hand, by detecting similarities in the dynamics of brain regions across different subjects, the proposed approach can detect characteristic fingerprints of a disease without the need of subsequent averaging the topological metrics; intersubject variability is thus incorporated as a feature of the analysis, as opposed to being a source of noise that has to be eliminated (Colclough et al., 2016; Hardmeier et al., 2014). While this makes more complex performing an analysis of the statistical significance of results, potential solutions include using null models obtained by mixing multiple conditions together, and analysing the stability of the topological metrics when these are calculated on random parts of the data (see Supporting Information).

At the same time, it is important to recognise two major limitations of the proposed methodology. First of all, the use of deep learning entails some computational challenges. Specifically, long enough time series have to be used, or alternatively a large number of trials per patients; in any case, enough data have to be available to avoid model overfitting (C. Zhang, Bengio, Hardt, Recht, & Vinyals, 2021). If large quantities of data cannot experimentally be secured, the researcher could resort to techniques to synthetically generate them, what is known as data augmentation (Taylor & Nitschke, 2018; Wen et al., 2020). Additionally, training DL models is computationally intensive, such that specific hardware acceleration is desirable in most applications.

Secondly, DL is inherently a black box approach, that is, it is challenging to explain why a set of data has been classified in a given way. In the context of this contribution, this implies that the model can tell us how similar the dynamics of two brain regions are, but not why. While many attempts have been made in recent years to extract intuitive and understandable components from DL models, no general solution has hitherto been proposed, and their application goes beyond the scope of this work (see for reviews on the topic Chakraborty et al., 2017; Ras, van Gerven, & Haselager, 2018); and Supporting Information for an occlusion analysis.

As with any proposal for a new method, the advantages and disadvantages will have to carefully be evaluated, especially in terms of the capacity for describing relevant brain functional structures, and differences between pathologies and conditions. This is especially important in the case of neuroscience, as available data are only high-level coarse-grained descriptions of the brain dynamics, and additionally the system cannot easily be intervened. Further analyses will therefore be needed, to confirm (or reject) the usefulness of both the concept of identifiability and the use of deep learning for its assessment, above and beyond what can be included in an initial study. Alternative ways of reconstructing functional networks based on the principle here discussed will have to be evaluated, for example, by comparing the change in identifiability between resting-state and cognitive tasks. Other deep learning models may also be considered, for example, those based on recurrent neural networks and long-short term memories (Smirnov & Nguifo, 2018). Finally, deep learning models can be substituted by any other model able to classify groups of time series, for example, by K-neighbors ones (Bagnall & Lines, 2014; Lee, Wei, Cheng, & Yang, 2012), or by classical machine learning models trained over sets of time series features (Christ, Braun, Neuffer, & Kempa-Liehr, 2018). We would nevertheless like to highlight a final point. Many works can be found in the literature, using deep learning as models to perform classifications (Al-Saegh et al., 2021; Craik et al., 2019). Deep learning nevertheless offers many more possibilities, especially when the classification score is used as a proxy to describe other aspects of the data, like the identifiability here discussed. The neuroscience community should therefore keep an open mind, and be aware of the usefulness of deep learning beyond its simplest and most direct applications.

## ACKNOWLEDGMENTS

M.Z. acknowledges CESGA (Supercomputing Centre of Galicia, Santiago de Compostela, Spain) for its supercomputing availability and support.

## SUPPORTING INFORMATION

Supporting information for this article is available at https://doi.org/10.1162/netn_a_00353.

## AUTHOR CONTRIBUTIONS

Massimiliano Zanin: Conceptualization; Formal analysis; Methodology; Visualization; Writing – original draft; Writing – review & editing. Tuba Aktürk: Data curation; Writing – original draft; Writing – review & editing. Ebru Yıldırım: Data curation; Writing – original draft; Writing – review & editing. Deniz Yerlikaya: Data curation; Writing – original draft; Writing – review & editing. Görsev Yener: Conceptualization; Data curation; Writing – original draft; Writing – review & editing. Bahar Güntekin: Conceptualization; Data curation; Supervision; Writing – original draft; Writing – review & editing.

## FUNDING INFORMATION

Massimiliano Zanin, H2020 European Research Council (https://dx.doi.org/10.13039/100010663), Award ID: 851255. Tuba Aktürk, Ebru Yıldırım, Deniz Yerlikaya, Görsev Yener, Bahar Güntekin, TÜBITAK, Award ID: 218S314. Massimiliano Zanin, Agencia Estatal de Investigación (https://dx.doi.org/10.13039/501100011033), Award ID: CEX2021-001164-M, funded by MCIN/AEI/10.13039/501100011033.

## Supplementary Material



## TECHNICAL TERMS

Functonal networks: Networks reconstructed by evaluating the statistical association between regions’ activation time series.
Deep learning: Family of machine learning algorithms based on processing data through artificial neural networks resembling brain circuits.
Activation function: Function that defines the output of an artificial neuron given the inputs it receives.
Convolution: Mathematical operation on two functions that expresses how the shape of one is modified by the other.
Overfitting: Training of a model that results in it learning too exactly the training data, and therefore failing to reliably generalise to future observations.
Hyper-parameter: Parameter of the model which is used to control the learning process, and that has to be defined prior to the execution of the task.
Surrogate time series: A synthetic time series constructed to preserve one or several statistical properties of the original series.
