# Supplementary material for: Reconstructing brain functional networks through identifiability and deep learning
Source: Netw Neurosci. 2024 Apr 1;8(1):241–59. doi: 10.1162/netn_a_00353 (PMC10923503; doi:10.1162/netn_a_00353)
Supplement: Supplementary file 1 [file netn-8-1-241-s001.pdf]

Supplementary Materials for

Reconstructing functional brain networks through identifiability and Deep Learning

Distribution of best DL models by channel

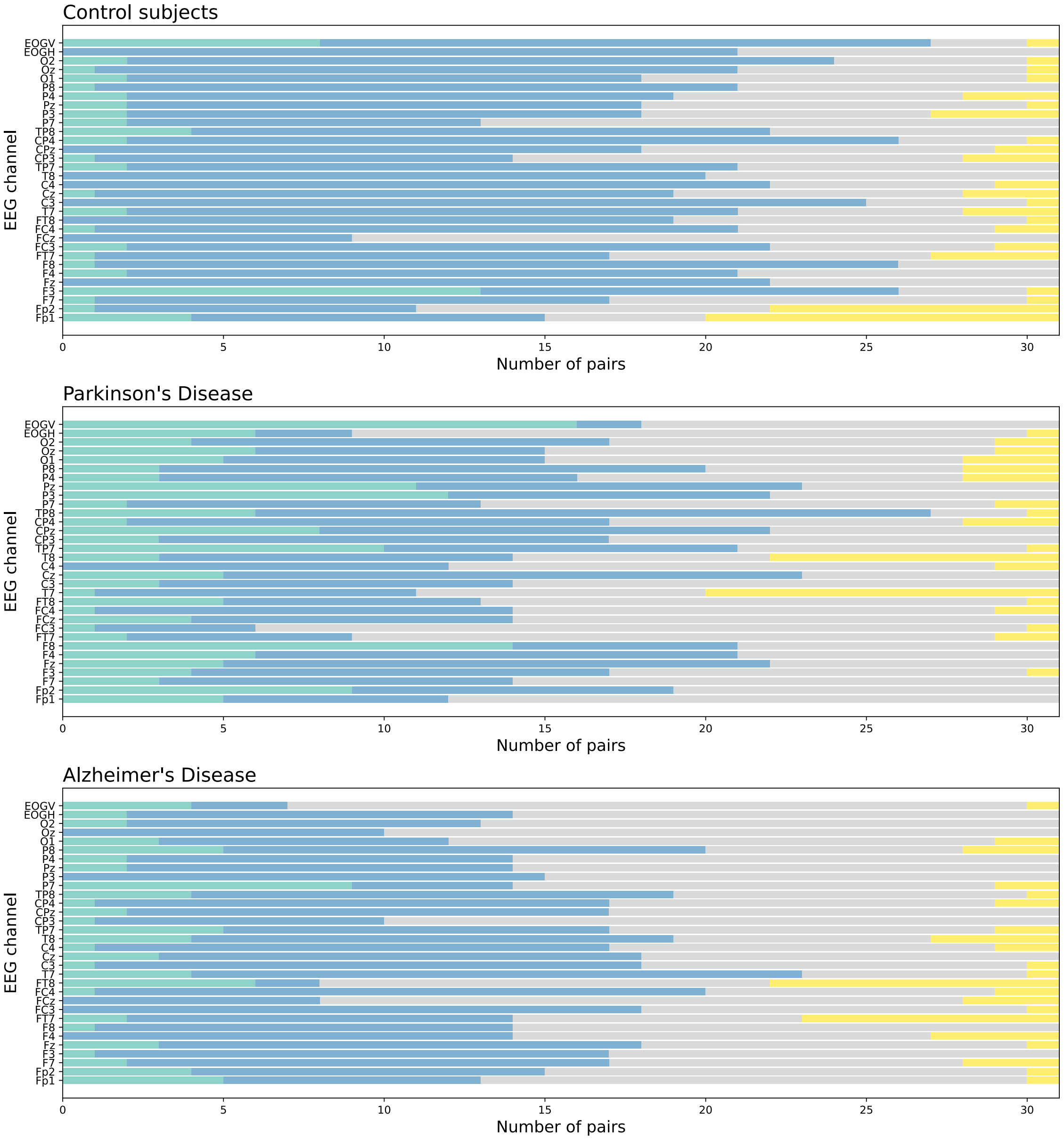

The figure depicts, for the three groups of subjects (control subjects, and Parkinson's and Alzheimer's Diseases patients), the number of times each model yielded the best classification score in each EEG channel. Colours are the same as in Fig. 1 of the main Manuscript.

Comparison of the four DL models

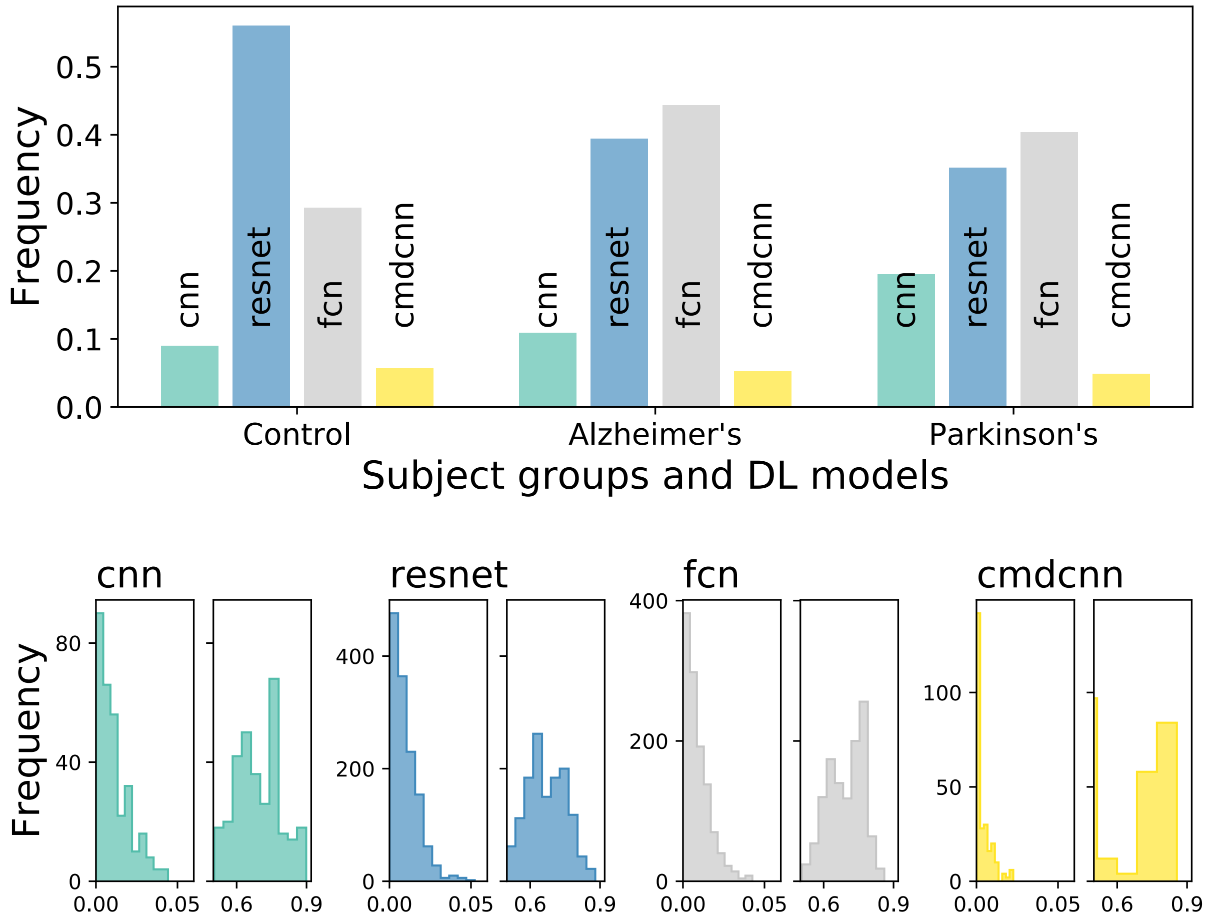

The top panel of the figure reports the number of time each model yielded the best result, as a function of the subject group. It can be seen that ResNet is the best performing model for the control group, but it is surpassed by FCN in the two patient groups - once again, highlighting the effect of the pathologies in the brain dynamics. The bottom panels report, for each model when that model yields the best result, the distance from the second best score (left subpanels) and the score itself (right subpanels). CMDCNN presents a different behaviour from the other models, as it achieves the best score only by small margins, and for very high classification scores; in other words, it outperforms the other models only when the differences between channels are evident.

Comparison of CNN and CMDCNN models

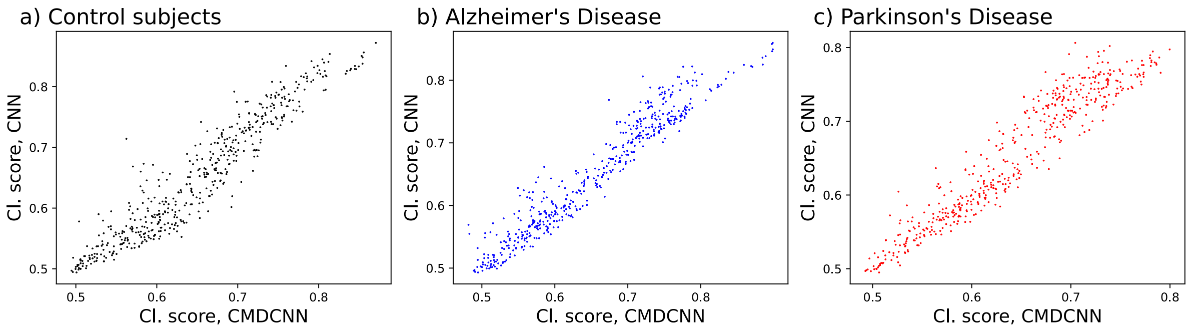

While CNN and CMDCNN are similar models based on the same inner operations, their different internal architecture implies that they do not always yield the same results. To illustrate this point, the previous figure depicts three scatter plots, each one corresponding to a different group of people, depicting the relationship between the classification scores obtained by both models. A clear correlation is observed, but also outliers, when one model outperforms the other one.

Changes in identifiability by condition

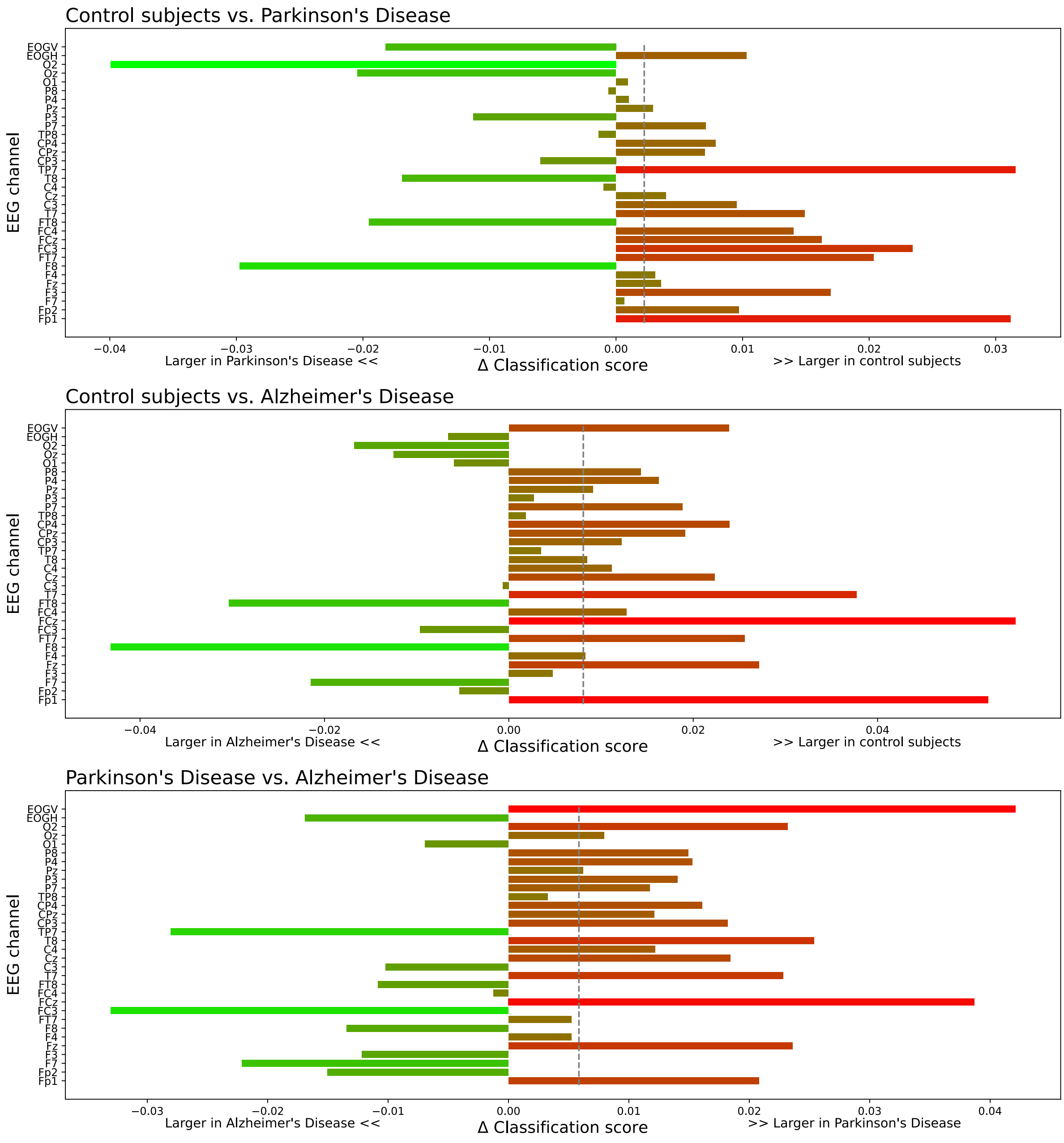

The three panels report the average difference in classification score, between the three considered conditions, organised by EEG channels. The dashed vertical lines indicate the average across all channels.

Classification score vs. Granger Causality and Transfer entropy

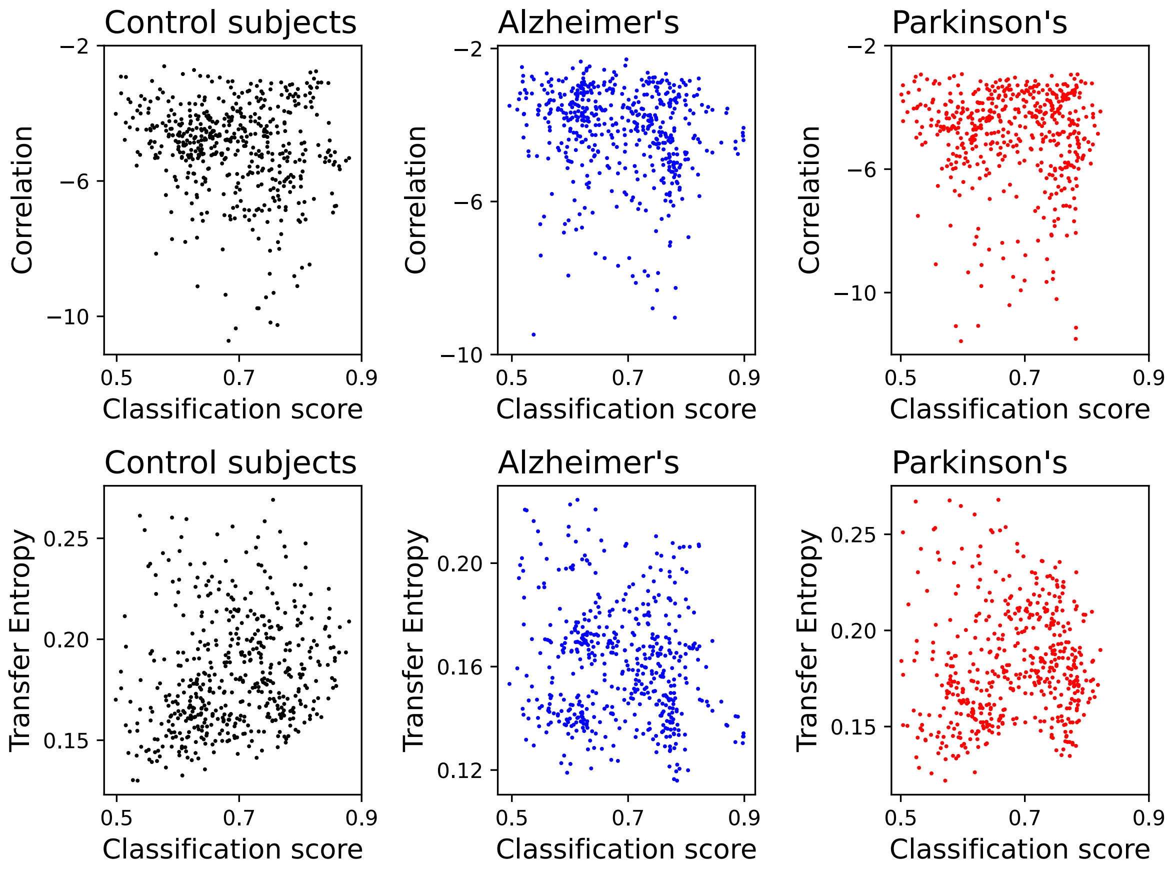

Top and bottom panels report scatter plots of the $\log_{10}$ of the p-value yielded by a Granger Causality test (top panels), and the results of calculating the Transfer Entropy (bottom panels) between pairs of channels, as a function of the corresponding classification score. From left to right, panels correspond to the three conditions here considered: control subjects (black dots), Alzheimer's Disease (blue dots), and Parkinson's Disease (red dots).

Classification score vs. physical distance between sensors

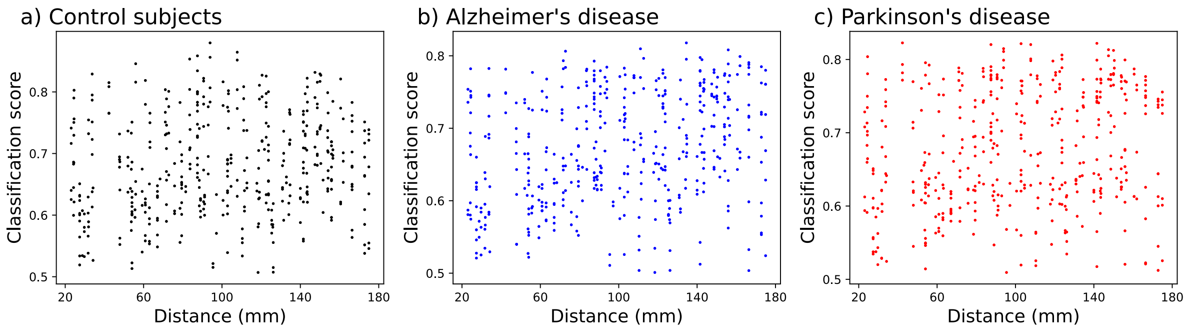

The three panels report scatter plots of the best classification score obtained for each pair of EEG sensors, as a function of their physical separation. Note that no clear relationship can be appreciated, suggesting that the cross-talk between sensors is not majorly affecting the classification task – i.e. even if two sensors record similar time series, differences may be enough to support the training of the DL model.

Topological metrics, networks reconstructed with the broadband signal

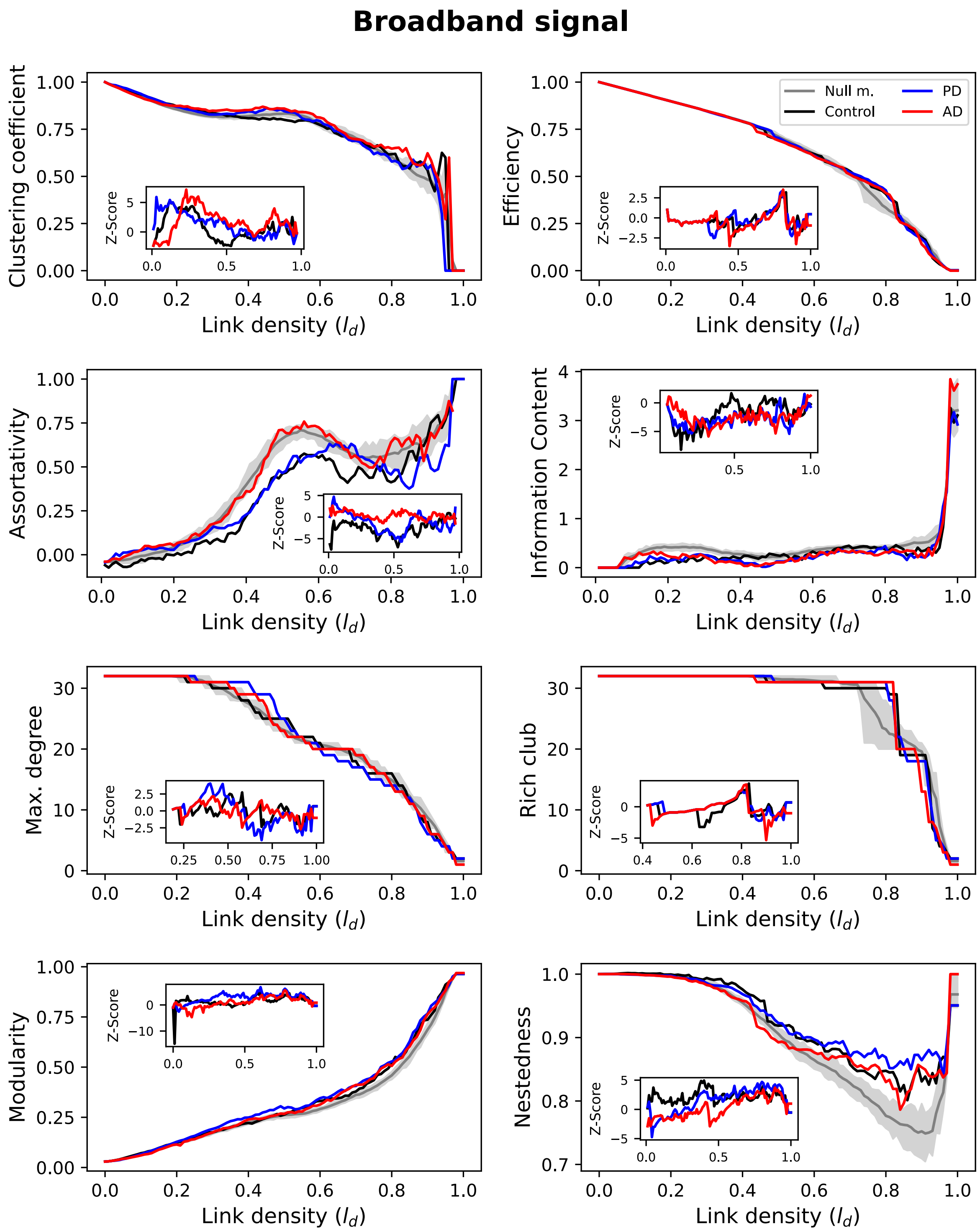

Topological metrics, alpha band

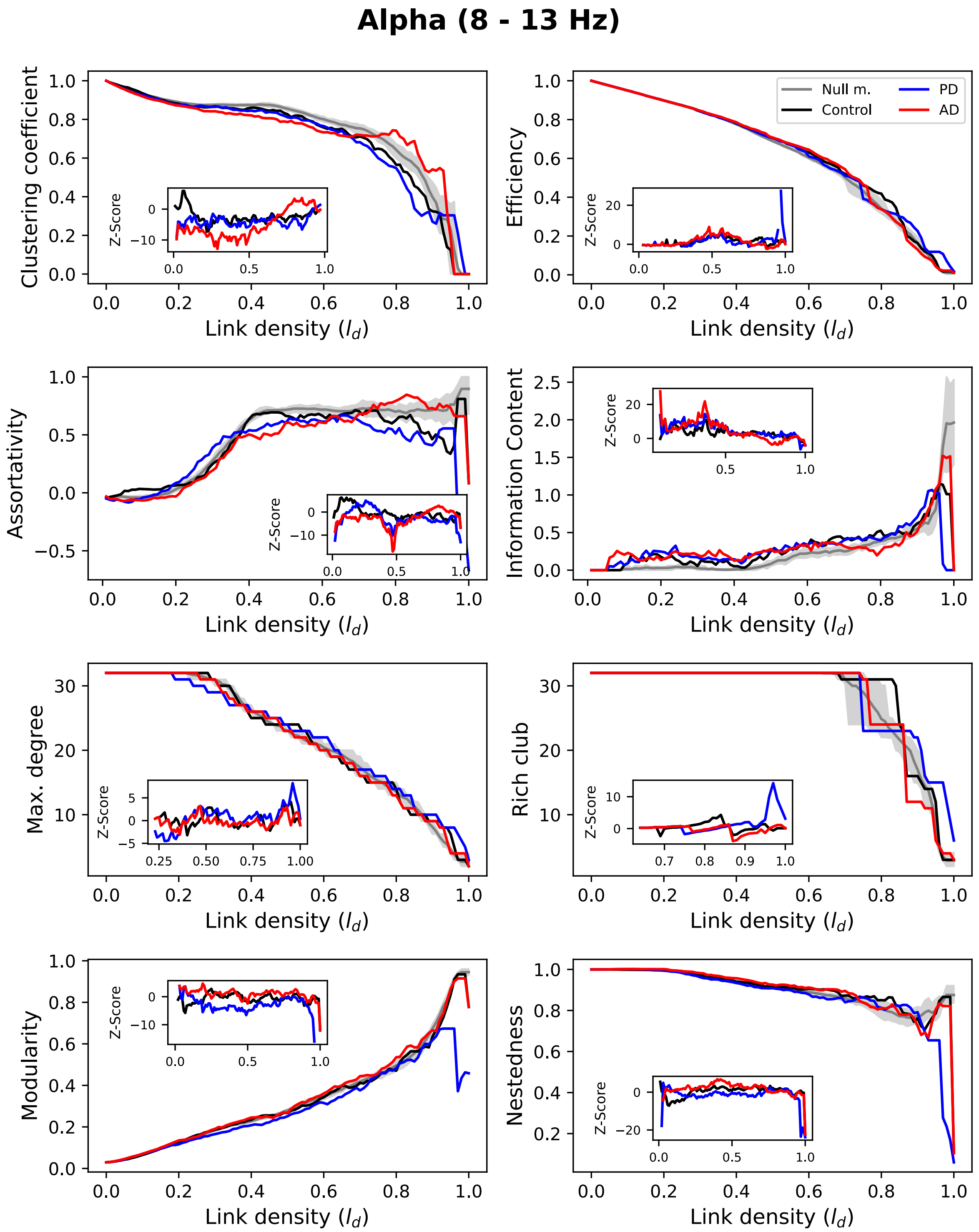

Topological metrics, beta 1 band

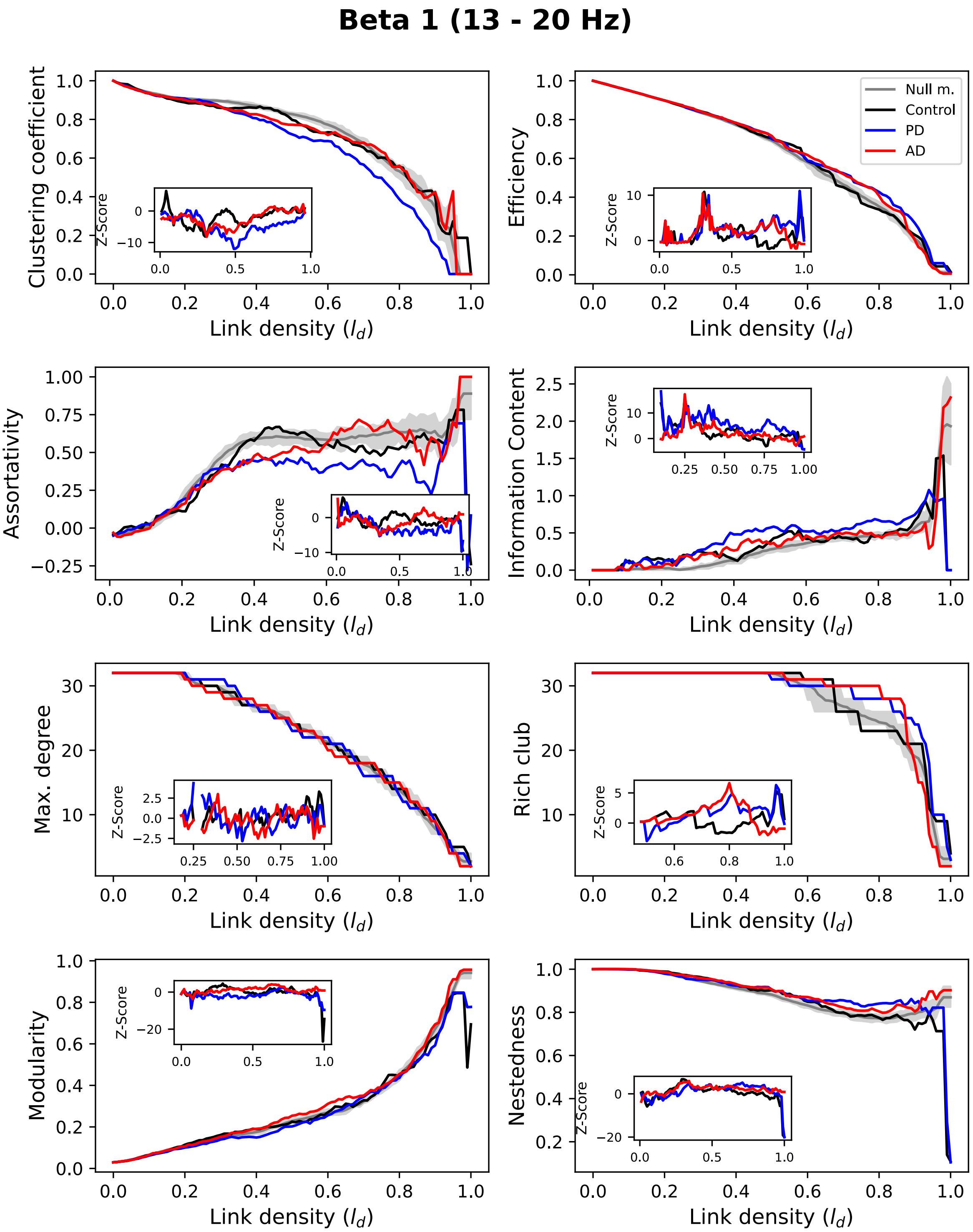

Topological metrics, beta 2 band

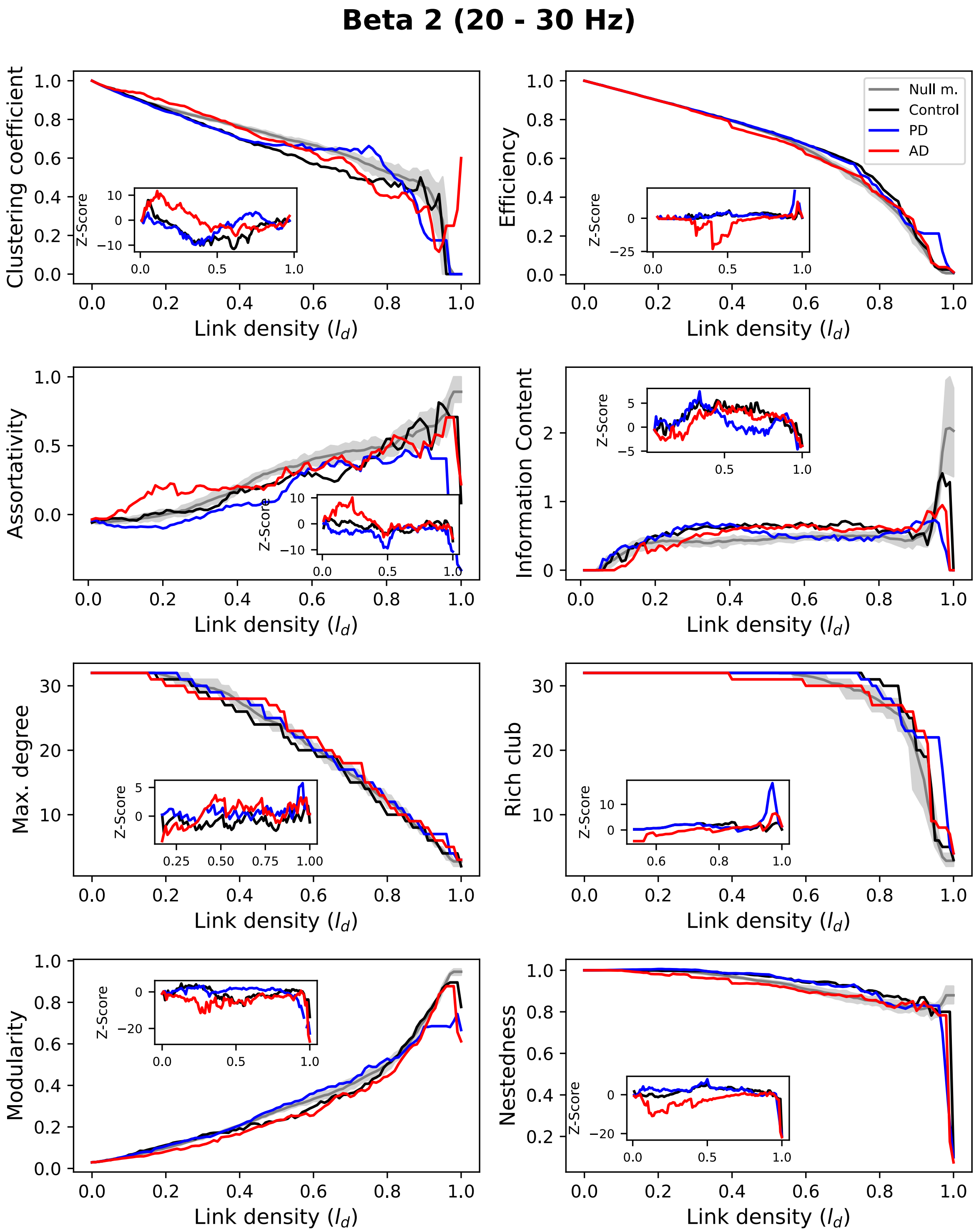

Topological metrics, gamma band

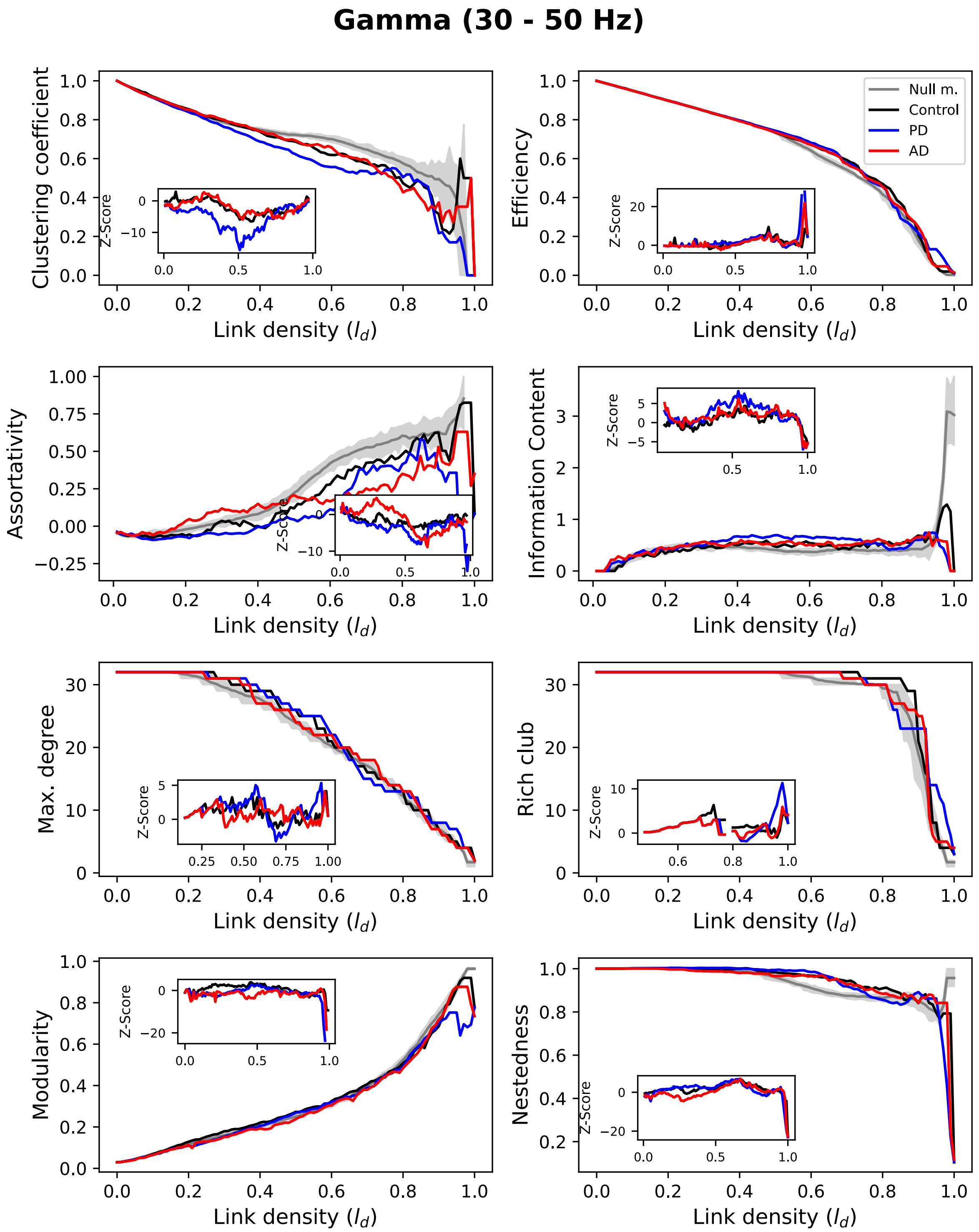

Stability of topological metrics, broadband signal

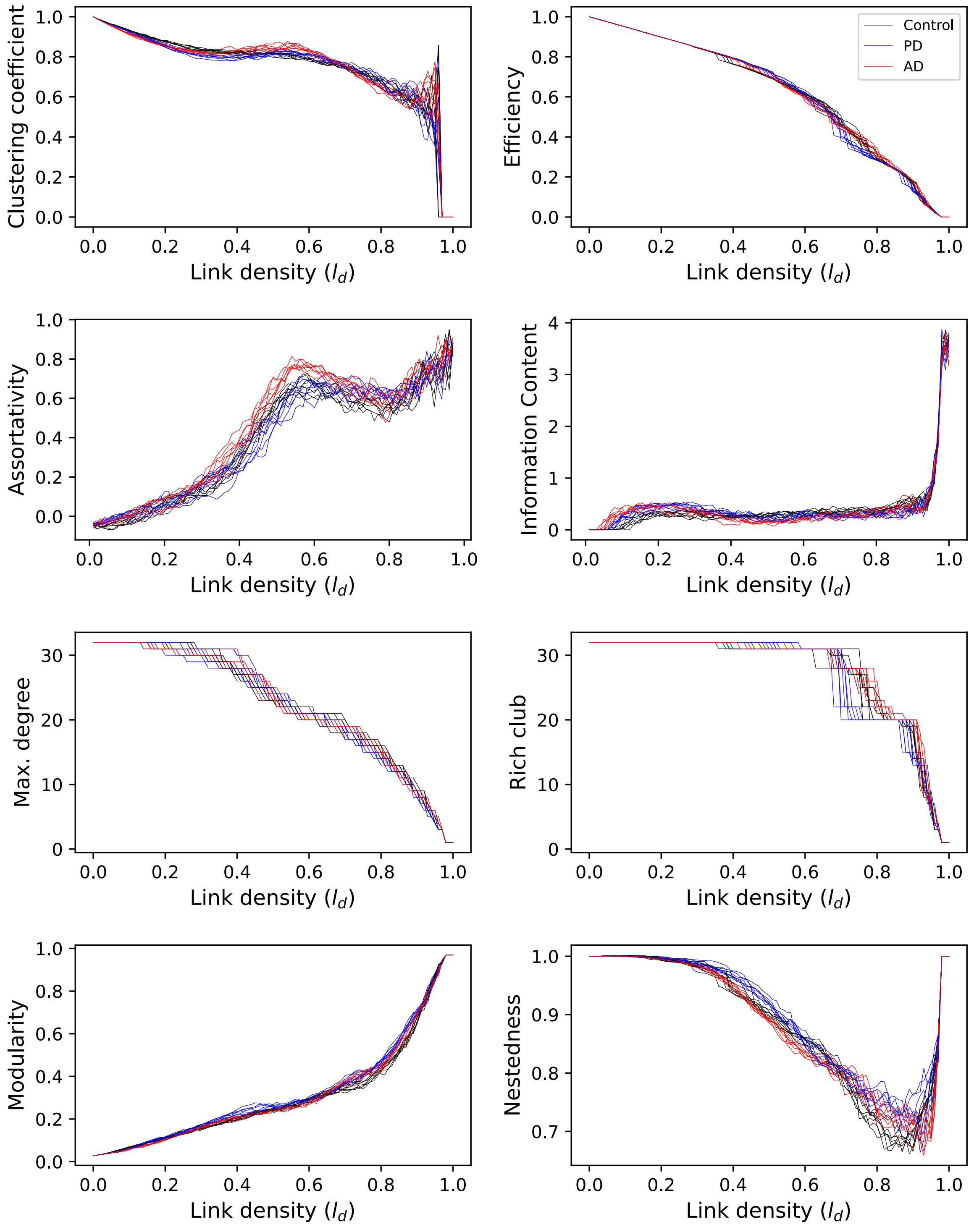

Evolution of the topological metrics, as a function of the link density, when networks are reconstructed using data corresponding to a random half of the subjects – each line corresponds to an independent realisation. Note that the differences between groups (mainly for AD in the clustering coefficient and the assortativity) are robust to the reduction in the size of the data set.

Topological metrics, broadband signal, eyes closed vs. open

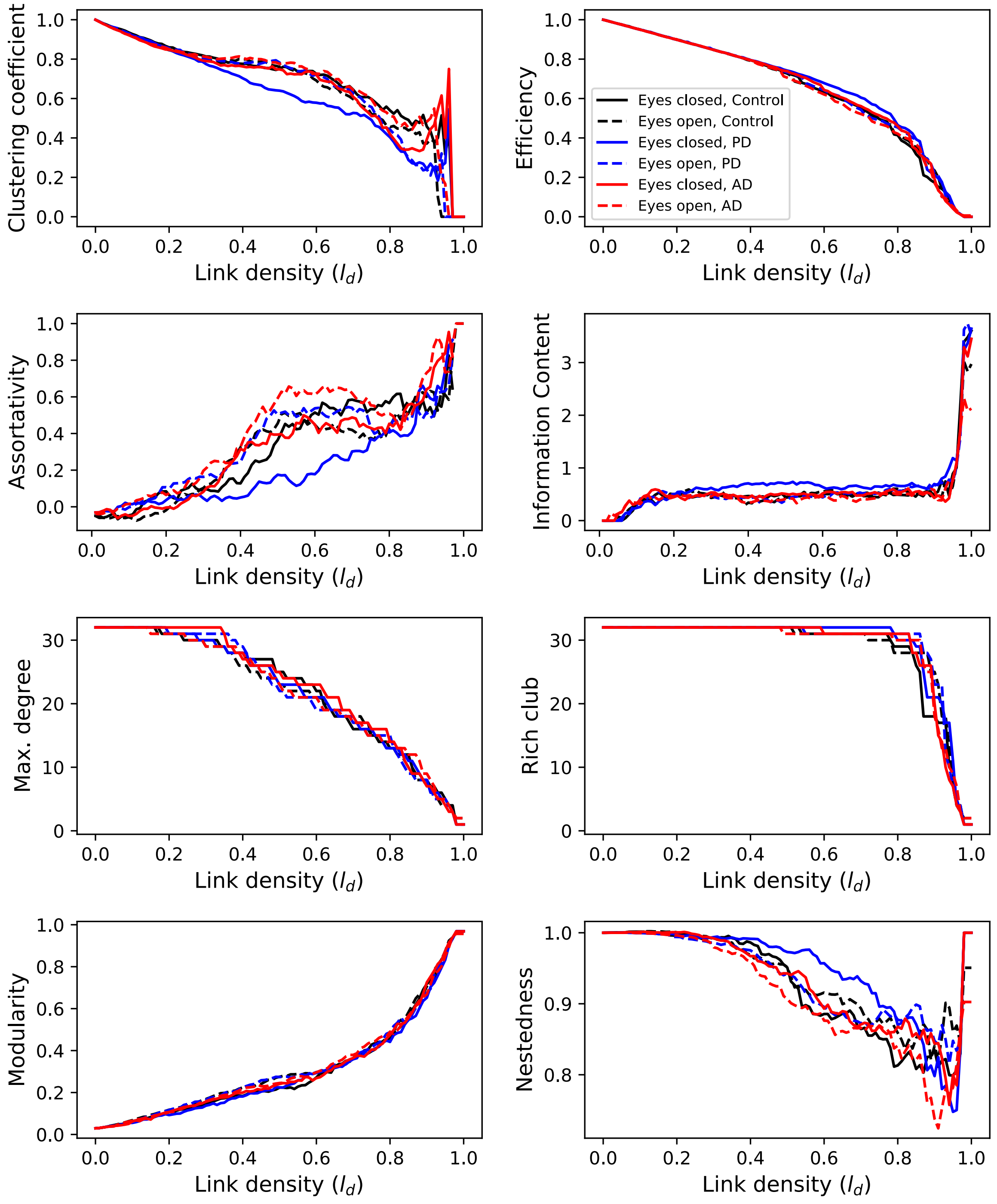

Z-Score of the topological metrics, broadband signal, eyes closed vs. open

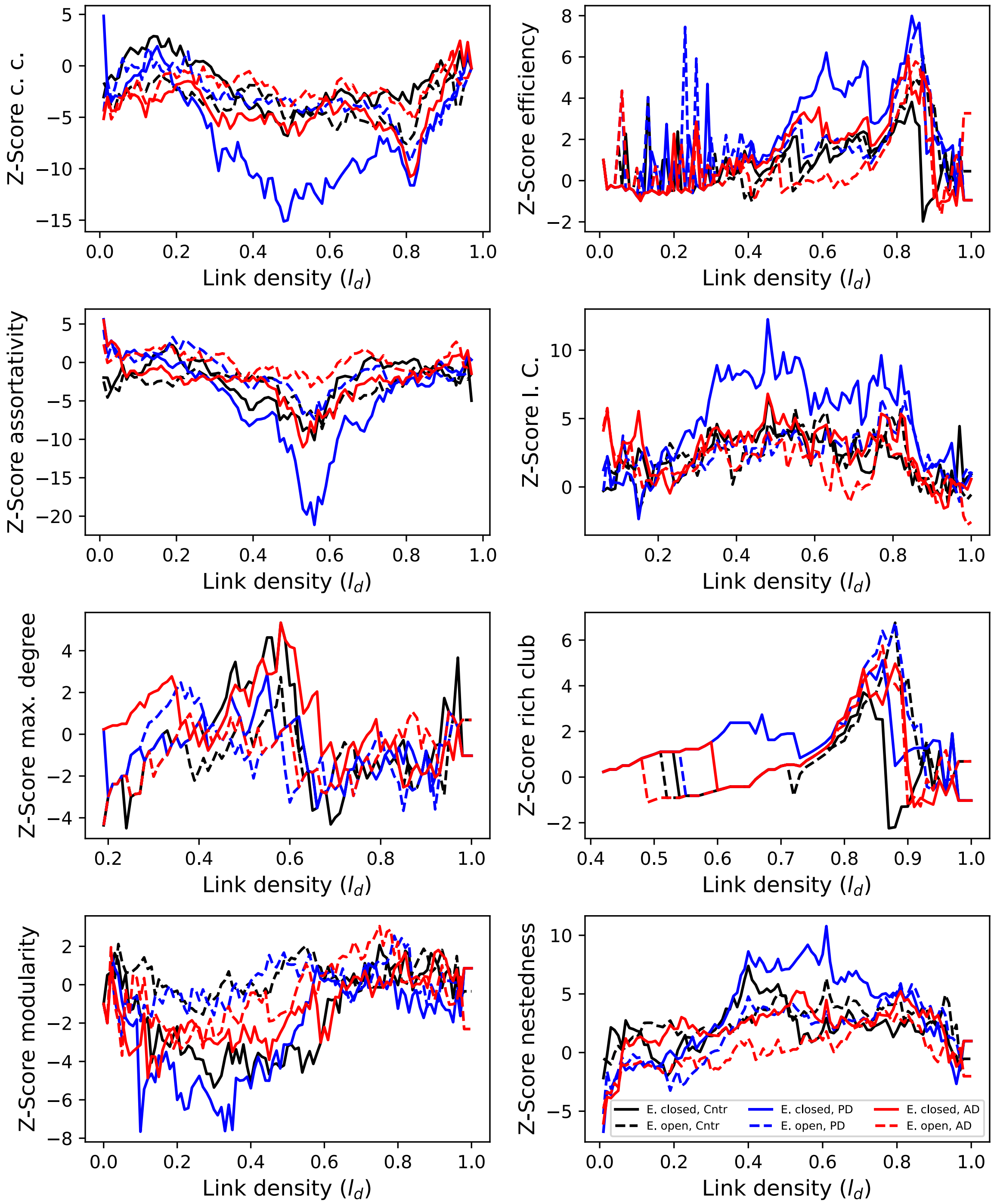

Channel identifiability, broadband signal, eyes closed vs. open

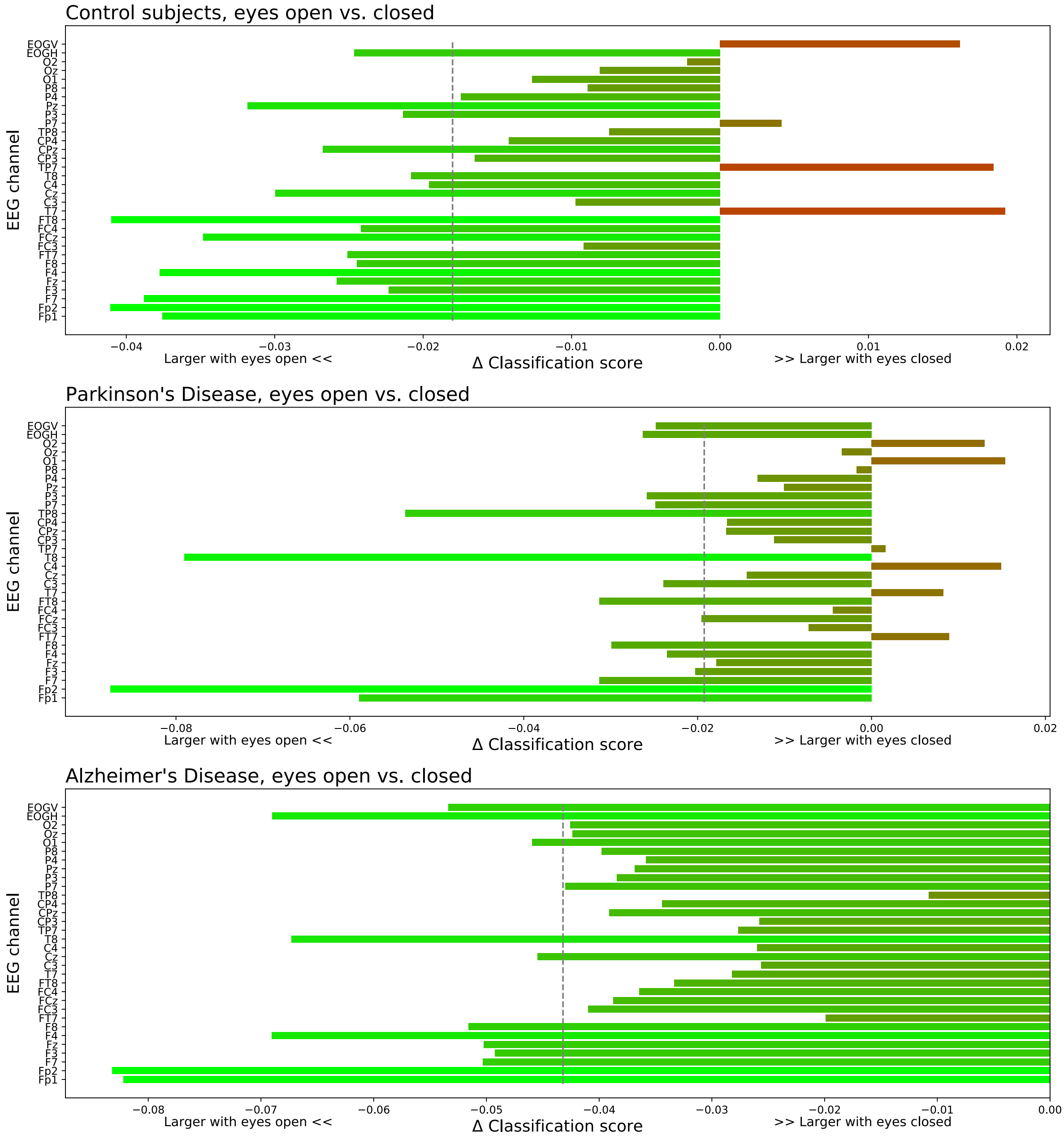

The three panels report the average difference in classification score (i.e. identifiability), comparing the results obtained in the classification when using time series corresponding to eyes open and closed conditions, organised by EEG channels. The dashed vertical lines indicate the average across all channels.

Classification score, true vs. surrogate time series

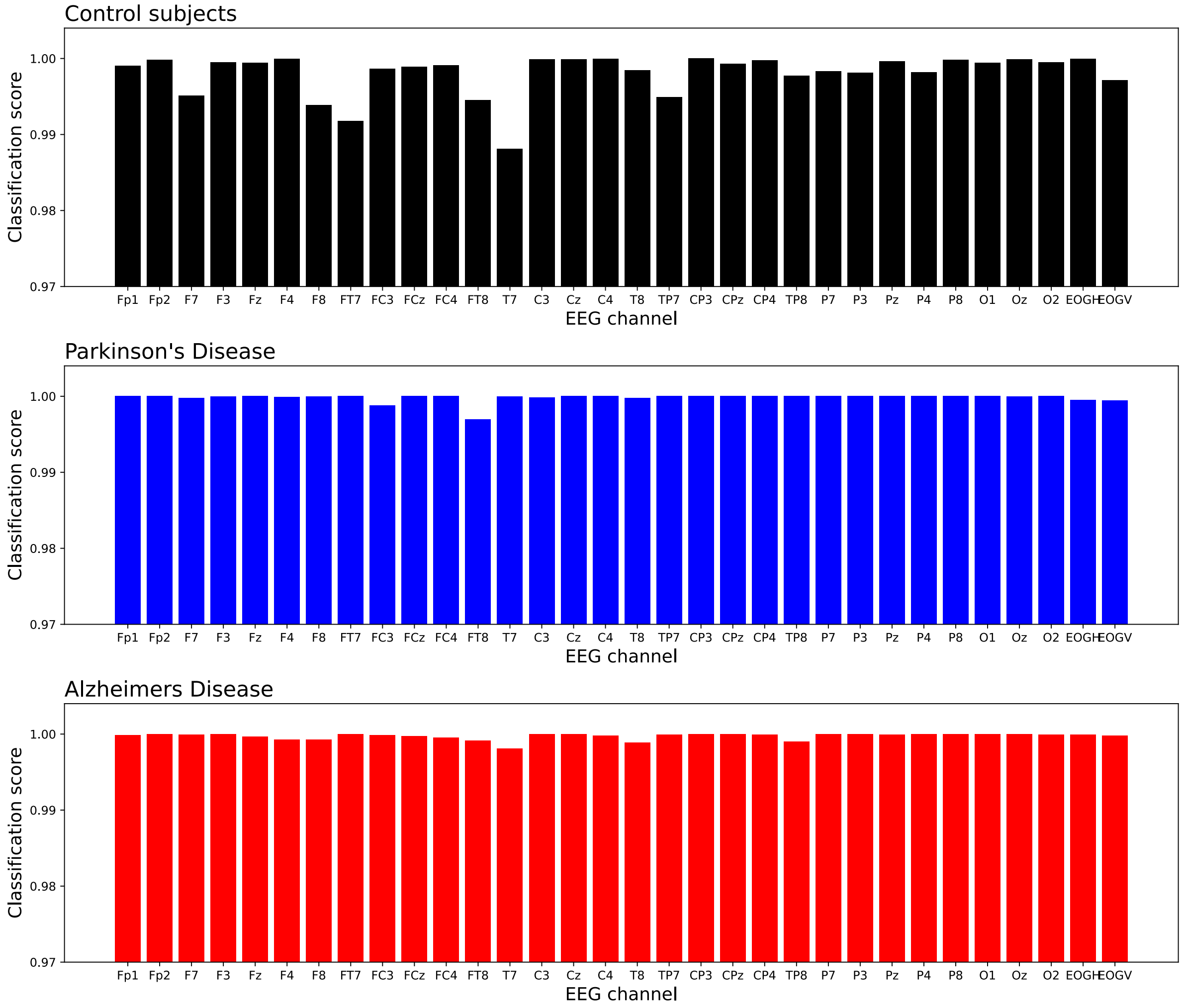

Average classification score for each patient group and EEG channel, obtained in a task of classifying between the true EEG recordings, and surrogate versions of the same time series. These surrogate versions are created by randomly shuffling the values of the time series, thus destroying temporal correlations while maintaining the amplitude distribution. All scores are very close to 1.0, supporting the hypothesis that the low classification score obtained for some pairs of EEG channels is due to a similar dynamics, and not to a random behaviour of the same.

Occlusion analysis

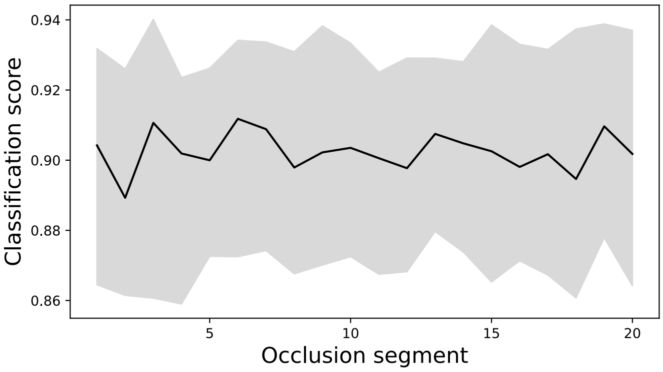

In order to check the possibility of extracting additional information from the DL models, this figure presents the results of an occlusion analysis. The original time series (here corresponding to the Fz and Cz channels of control subjects) have been divided into 20 segments; elements of each one of them has been set to zero in each iteration, and a classification task has then been performed. If one of those segments would contain relevant information, the corresponding classification score would present a substantial drop.

Results (average over 20 realisations, solid line, and 10-90 percentile band) indicate that this is not the case, and that all segments are equally important. This is to be expected, as time series are extracted randomly from the complete EEG recordings; hence, nothing special defines their beginning and end points.

Analysis of the time series length

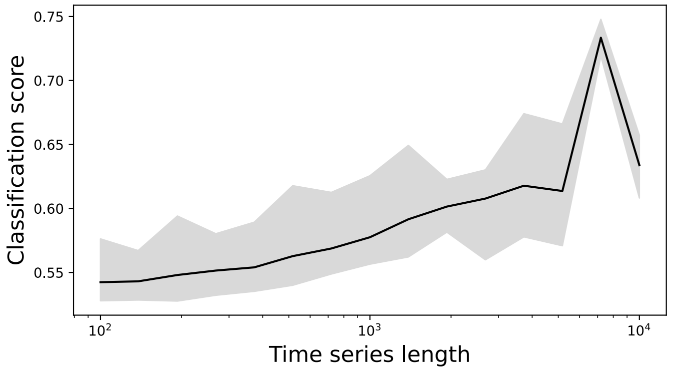

We here present an analysis of the effect of the length of the considered time series in the classification task. The original time series (here corresponding to the Fz and Cz channels of control subjects) have been divided in segments of different length, and then a classification process has been performed; the solid black line in the figure above depicts the evolution of the classification score as a function of such length, with the grey band representing the 10-90 percentile over 100 realisations.

Note that the choice of this length is a non-trivial process, that has to balance the need of long-enough time series to ensure sufficient information is included in them to achieve a meaningful classification; and the risk of disregarding the non-stationarity of the signal when segments are too long. At the same time, the chosen length also affects the number of available segments, and hence the reliability of the DL model training.

While it may be tempting to choose the length yielding the highest classification score, other aspects have to be taken into account. Specifically, a higher classification score does not imply that the reconstructed networks are more representative; note that these are invariant under a monotonous transformation of the score. Additionally, one may be interested in transient patterns in the time series, even if they make the classification more challenging.

A full analysis of the consequences of different choices of the segment's length on the reconstructed topological metrics, while interesting, is beyond the scope of this work.
